# Monitoring vibronic coherences and molecular aromaticity in photoexcited cyclooctatetraene with an X-ray probe: a simulation study†

**DOI:** 10.1039/d2sc04335a

**Published:** 2023-02-14

**Authors:** Yeonsig Nam, Huajing Song, Victor M. Freixas, Daniel Keefer, Sebastian Fernandez-Alberti, Jin Yong Lee, Marco Garavelli, Sergei Tretiak, Shaul Mukamel

**Affiliations:** a Department of Chemistry, University of California Irvine California 92697-2025 USA yeonsig@uci.edu smukamel@uci.edu; b Physics and Chemistry of Materials, Theoretical Division, Los Alamos National Laboratory Los Alamos New Mexico 87545 USA; c Departamento de Ciencia y Tecnologia, Universidad Nacional de Quilmes/CONICET B1876BXD Bernal Argentina; d Department of Chemistry, Sungkyunkwan University Suwon 16419 Korea jinylee@skku.edu; e Dipartimento di Chimica Industriale “Toso Montanari,”, Universita‘ degli Studi di Bologna I-40136 Bologna Italy

## Abstract

Understanding conical intersection (CI) dynamics and subsequent conformational changes is key for exploring and controlling photo-reactions in aromatic molecules. Monitoring of their time-resolved dynamics remains a formidable experimental challenge. In this study, we simulate the photoinduced S_3_ to S_1_ non-adiabatic dynamics of cyclooctatetraene (COT), involving multiple CIs with relaxation times in good agreement with experiment. We further investigate the possibility to directly probe the CI passages in COT by off-resonant X-ray Raman spectroscopy (TRUECARS) and time-resolved X-ray diffraction (TRXD). We find that these signals sensitively monitor key chemical features during the ultrafast dynamics. First, we distinguish two CIs by using TRUECARS signals with their appearances at different Raman shifts. Second, we demonstrate that TRXD, where X-ray photons scatter off electron densities, can resolve ultrafast changes in the aromaticity of COT. It can further distinguish between planar and non-planar geometries explored during the dynamics, as *e.g.* two different tetraradical-type CIs. The knowledge gained from these measurements can give unique insight into fundamental chemical properties that dynamically change during non-adiabatic passages.

## Abstract 2

Monitoring conical intersection and aromaticity changes in photo-relaxation of cyclooctatetraene by TRUECARS and TRXD.

## Graphical abstract

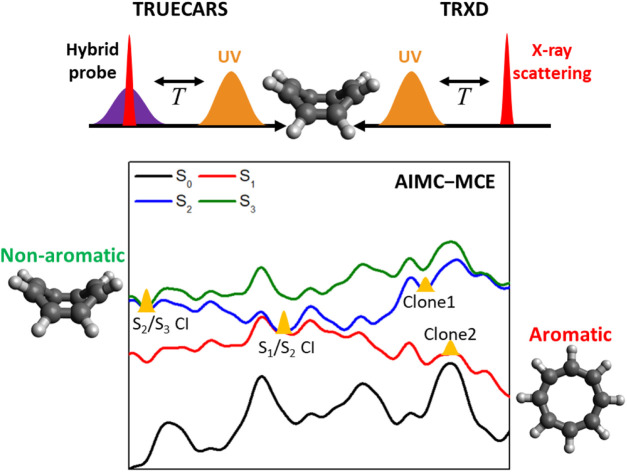

## 1 Introduction

Aromaticity is a property of cyclic (ring-shaped), typically planar (flat) molecular structures with delocalized π electrons that gives increased stability compared to saturated (non-aromatic) compounds having single bonds or other non-cyclic arrangements with the same set of atoms. Aromaticity plays key roles in chemical reactions (electrophilic aromatic substitution^1^), molecular physics (organic semiconductors^2^ and aromatic ring currents^3^), and biochemistry, where amino acids serve as building blocks of proteins. Thus, monitoring conical intersection (CI) dynamics and conformational changes is key for unravelling and controlling photochemical reactions in aromatic molecules.

Cyclooctatetraene (COT) is a conjugated cyclic 4*n* π-electron system that has a *D*_8h_ planar conjugated aromatic π-network in the lowest excited state but is non-aromatic in higher excited states (S_*n*>1_) as well as in the ground state, where it has a non-planar boat-like *D*_2d_ structure with localized single and double C–C bonds. Thus, COT may serve as a prototypical photoactive unit where photon absorption is employed as a control knob to switch between non-aromatic and aromatic states. Therefore, photorelaxation through CIs induces strong modification of the planarity and electron density, and thereby aromaticity, but without ring opening like heterocyclic compounds.^4,5^

**Scheme 1:**
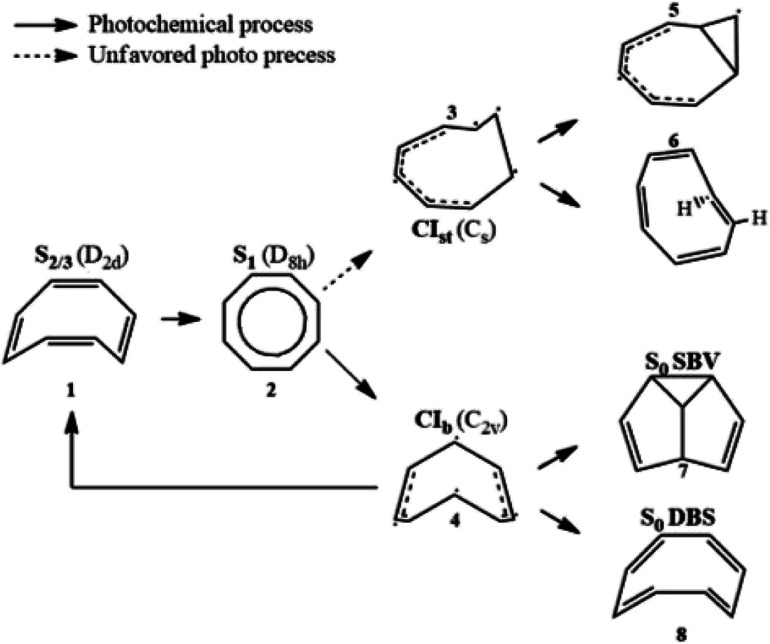
Photochemical and thermal reaction scheme for cyclooctatetraene.

Its thermal and photochemical relaxation pathways have drawn significant experimental and theoretical attention^6–8^ (Scheme 1). A photon initially excites COT to the optically allowed (bright) S_2/3_ state (1), followed by ultrafast non-radiative decay to the optically forbidden (dark) S_1_ state (2). A non-adiabatic transition to S_0_ is controlled by two tetraradical-type conical intersections (CI),^8,9^ CI_st_ (3) and CI_b_ (4). CI_st_ has a typical out-of-plane triangular –(CH)_3_– kink of a triradical nature similar to other unsaturated hydrocarbons,^10,11^ leading to three- or four-membered ring formation (5) and *cis* → *trans* isomerization (6).^8^ However, the decay *via* this channel is suppressed by an energy barrier. CI_b_ holds C_2v_ symmetry and has two unpaired electrons centered at single carbon atoms and two resonance-stabilized allyl radicals.^9^ Thanks to its lower barrier, it acts as an elective radiation-less channel leading to the formation of semibullvalene (SBV, 7) as a main product,^12^ and a double-bond shifted (DBS, 8) or the original COT (1) is formed as byproducts.^13^ We refer to ref. ^8^ for the comprehensive photo/thermal reaction pathways.

The DBS yields a degenerate product of the parent COT, where the π-electrons migrate within the octagonal perimeter, resulting in a site exchange between singly- and doubly-bonded carbon atoms eventually leading to photoinduced bond order inversion within the ring. Modulation of these pathways in favor of desired channels could be accomplished by chemical modification with π-donor and -acceptor substituents, or by quantum control strategies, making COT a potent and attractive design unit for *e.g.* molecular photoswitches that allow control of aromaticity and bond order.^14,15^

While the S_1_ to S_0_ relaxation pathway has been extensively studied, the nonradiative S_3_ to S_1_ decay has remained largely unexplored. It is a key issue prior to unravelling the role of S_1_/S_0_ CI, since the dark S_1_ state is only accessible by relaxation from the higher excited states. The S_3_ → S_1_ relaxation can further affect the nuclear dynamics on S_1_, thus influencing the resulting photoproducts. CIs play important roles in many photophysical and photochemical processes.^16,17^ Monitoring CI pathways is important for controlling and achieving the desired photoproducts. Various studies have shown that modifying the initial condition of nuclear wavepackets in the electronic states forming the CIs can have tremendous effects on the photoproducts,^18,19^ corroborating the necessity of including the S_3_ → S_1_ relaxation mechanism in photoexcited COT.

Thanks to the unique temporal, spectral and spatial resolutions provided by free-electron X-ray light sources, many X-ray techniques have been proposed to monitor the CIs in molecules.^20,21^ A popular technique is femto/attosecond spectroscopy, which indirectly probes CIs by depletion/appearance/bifurcation of the absorptive lines.^22–24^ Polli *et al.* implemented ultrafast optical spectroscopy to probe light-induced photoisomerization of rhodopsin and mapped out the energy gap between the ground and excited electronic states as a function of time.^25^ Such evidence is indirect and circumstantial and does not give the direct signature of CI.

To this aim, transient redistribution of ultrafast electronic coherences in attosecond Raman signals (TRUECARS) has been theoretically proposed.^26^ This technique directly monitors vibronic coherences created during the CI passage with no contributions of populations (background-free), which is more direct evidence or which signifies the presence of CI better than the energy gap between the involved states. A hybrid broadband/narrowband pulse used in TRUECARS can offer a good combination of both spectral and temporal resolutions. This stems from the ultrafast timing of CIs as well as the few tens of eV energy range spanned by the vibronic coherences. It has been theoretically used to monitor the CI passage in photo-relaxation in (4-thio)uracil^27–29^ and energy transfer in a heterodimer^30^ and a triarylamine trimer.^31^ A major difficulty in the implementation of TRUECARS is the precise phase control between two pulses, which is under development. Herein, we focus on how a TRUECARS signal distinguishes two different CI passages during photorelaxation in COT.

On the other hand, time-evolving electronic charge densities ain CI passages can be imaged with subfemtosecond resolution using ultrafast time-resolved X-ray diffraction/scattering (TRXD).^32^ In ultrafast TRXD experiments, a molecule is prepared in a time-evolving superposition of states by using an optical laser, undergoing non-stationary dynamics, and then a hard X-ray probe pulse is scattered by the excited molecule onto a detector, yielding the three components of the scattering signal: elastic scattering, inelastic scattering, and mixed (in)elastic scattering related to electronic coherence which contribute to the signal.^32,33^ The snapshots at different pump-probe delays create a movie presenting temporal evolution of electron densities triggered by the pump pulse. Since the pioneering theoretical work of Wilson *et al.*,^32^ there has been an immense development of theory^34–37^ to study non-stationary molecular samples in excited electronic states. A novel development of bright XFELs extended the X-ray scattering measurement in a solid crystal into gas or liquid phases,^38–40^ which involves real-time monitoring of the coherent vibrational motion of excited *N*-methylmorpholine^41^ and its orientation of the transition dipole moment using gas-phase X-ray scattering.^42^ In the liquid phase, ultrafast hydrogen bond dynamics,^43^ solvent reorganization coupled to intramolecular charge transfer,^44^ liquid–liquid phase transition,^45^ and structural changes of proteins^46^ have been monitored. Nielsen *et al.* recorded coherent nuclear dynamics with atomistic resolution on the excited^47^ and ground state^48^ potential energy surfaces for systems in an environment. XFELs are being upgraded to achieve brighter light sources, higher repetition rate, and greater spatiotemporal resolution to expand their applications.

Our previous theoretical work has demonstrated that the TRXD signals can potentially image transient electron transition densities directly associated with CI passages in azobenzene^49,50^ and 4-thiouracil,^29^ exhibiting characteristic positive/negative oscillations due to the formation of electronic coherences. We had further shown that the two-dimensional diffraction pattern, dominated by elastic scattering, can be used to monitor conformational changes *e.g.*, *cis* to *trans* photoisomerization in azobenzene.^49^ Such valence electron densities can be used to monitor the electron density (aromaticity) and planarity variation of the molecule.

In the present study, we implement a non-adiabatic excited state molecular dynamics^51^ protocol to track the S_3_ → S_1_ relaxation pathway in optically excited COT. We employ a semiempirical *ab initio* multiple cloning (AIMC) approach based on Multi-Configurational Ehrenfest (MCE), which provides an accurate description of non-adiabatic molecular dynamics in large conjugated molecules^52^ with affordable cost.

We capture the appearance of vibronic coherences as well as aromaticity changes upon photorelaxation by using an ultrafast X-ray probe. The vibronic coherences generated at the multiple CIs are tracked by the TRUECARS signal. We show highly diverse scenarios for excited state relaxation and record the temporal and the energetic profiles of CIs by using the TRUECARS signal and its spectrogram. We find that explicit use of transition polarizabilities is crucial to assess accurate observation of vibronic coherences evolving during the CI passage. Due to the quantum nature of nuclear motions, the vibronic coherences do not vanish after passing through the CI passage. The TRUECARS signal provides a clear signature of the two CIs (S_3_/S_2_*vs.* S_2_/S_1_) by their different timings and energy splitting distributions between the involved states.

We find that TRXD is a powerful tool for the real-time tracking of the aromaticity and molecular conformation changes in molecules by tracking the evolving valence electron densities. The 2D elastic scattering pattern can differentiate the different CI pathways, CI_st_ and CI_b_, and the photoproduct, SBV from the reactant COT. This helps map the comprehensive relaxation pathways of COT from bright S_3_ to S_0_*via* the dark and aromatic S_1_ state.

## 2 Results and discussion

The AIMC approach was employed to describe the S_3_ → S_1_ electronic transitions in COT. AIMC naturally includes decoherence through cloning events when mean-field theory fails to describe two electronic states evolving on very different surfaces. This approach has been successfully applied to describe photoinduced dynamics in large molecules, such as a dendrimer^31^ and a bichromic molecule.^30^

**Fig. 1:**
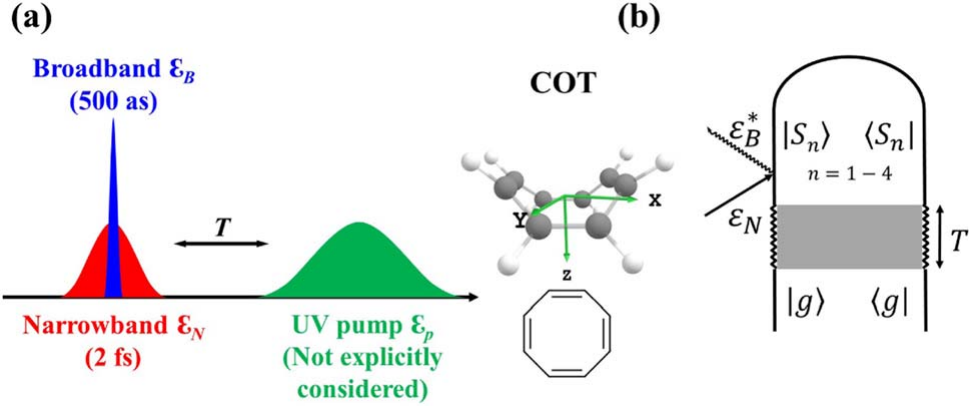
(a) Pulse configuration and (b) loop diagram for TRUECARS with cyclooctatetraene (COT) placed on the *xy* plane (Lewis structure given). The grey area indicates the electronic and nuclear population in the bright S_3_ state created by the pump pulse 
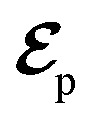
 (not considered explicitly in the simulation) and a free evolution period of the molecule. At time delay *T*, the hybrid 
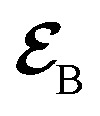
 (broad) and 
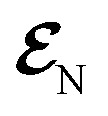
 (narrow) pulse is applied to probe the dynamics. See the ESI† for loop diagram rules.

The vibronic coherences emerging at the excited state CIs are tracked by the TRUECARS signal and the geometry and aromaticity changes are monitored by the TRXD signal. TRUECARS uses a hybrid field 
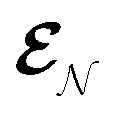
 (2 fs), with a central frequency of 200 eV and 
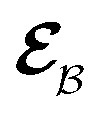
 (500 as) following the pump-probe waiting time *T* (Fig. 1). The central frequency was chosen to maximize the signal strength by maximizing the polarizability cross section while still staying off-resonant (pre-resonant).^53^ Otherwise the populations will contribute and dominate the coherences.^54,55^ The signal is finite only when there is an overlap of nuclear wavepackets in different electronic states, making this technique free from population background. A single broadband pulse with the same central frequency and bandwidth (Fig. S1†) is used for TRXD.

**Fig. 2:**
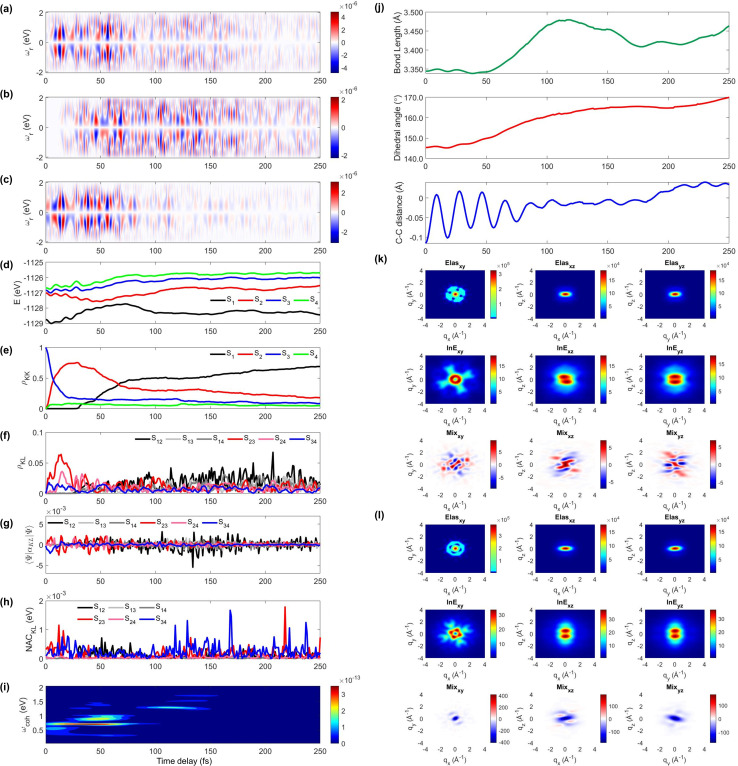
TRUECARS and TRXD signals and relevant molecular properties in the ensemble of 98/57 trajectories of COT. The averaged frequency-dispersed TRUECARS signal *S*(*ω*_r_, *T*), for (a) total, (b) S_2_/S_1_ coherence, and (c) S_3_/S_2_ coherence. (d) Combined potential energy surfaces of the electronic states in all trajectories. (e) Averaged population in the electronic states. (f) Averaged coherence magnitude *ρ*_*KL*_ between electronic states, according to eqn (3). (g) Averaged expectation value of the polarizability operator calculated with eqn (5). (h) Averaged nonadiabatic coupling magnitude for each coherence *ρ*_*KL*_. (i) Averaged FROG spectrogram, according to eqn (7), which is extracted from the TRUECARS signal by integration over the negative Raman shifts (*ω*_r_ < 0). (j) Time evolution of the averaged molecular geometry, bond length (top), dihedral angle (C_1_–C_4_ in Fig. S6a†), and bond alternation (bottom). (k) Averaged two-dimensional TRXD scattering pattern projected on the *xy* (left), *xz* (middle), and *yz* (right) planes at *T* = 1 fs. The top, middle, and bottom panel shows contributions from elastic scattering, inelastic scattering, and mixed elastic/inelastic scattering (coherence) to the total signal, respectively. (l) Same as (k) but at *T* = 250 fs.

We display the TRUECARS/TRXD signal as well as evolution of population, coherence, and molecular geometry, averaged over the total 98/57 trajectories with equal weight, in Fig. 2. Note that only the signal averaged over all trajectories is experimentally observable although individual trajectories illustrate different molecular dynamics scenarios. We first note that our AIMC approach well describes the S_3_ → S_1_ photo-relaxation dynamics of COT. The S_1_ population dynamics (Fig. 2e) is fitted with *P*(*t*) = *A*e^−(*t*−*t*_0_)/*k*^, with *t*_0_ = 26.5 fs, yielding a growth time of 54.5 fs. This is in a good agreement with our previous surface hopping simulations^56^ and time-resolved mass spectroscopy measurement of photoexcited cyclooctatriene and bicyclooctadiene using a near-IR photoionization probe,^57^ where ^1^*B*_2_ to ^2^*A*_1_ (*C*_2*v*_ symmetry, corresponding to S_3_ to S_1_ in the current study) relaxation was estimated to occur within 67 fs. The average population is distributed with a large fraction (70%) in S_1_ and a smaller one in S_2_ (20%) and the others for S_3_ to S_4_ states at 250 fs.

The TRUECARS signal (Eqn (4) and (5)) is visible over the entire simulation time, with stronger magnitudes at *e.g.* 0 to 70 fs. The two CI passages are well captured by the TRUECARS signal (S_2_/S_1_ in Fig. 2b and S_3_/S_2_ in Fig. 2c). The molecule enters the S_3_/S_2_ CI region, with strong nonadiabatic coupling (NAC) (Fig. 2h) due to their close spacing in energy (Fig. 2d), creating a vibronic coherence (Fig. 2f), and thereby, the TRUECARS signal shows up from the beginning and maintains its amplitude until 70 fs (Fig. 2c). A delayed S_2_/S_1_ CI is observed (Fig. 2b) as the major population transfer occurs between 50 and 100 fs, but the relevant TRUECARS signal remains strong until 170 fs. We note that after the second CI, S_2_ and S_1_ evolve differently, and their energy splitting increases, as the TRUECARS signal is observed at a higher Raman shift *ω*_r_ (Fig. 2b). We find that the S_4_ state is only slightly affected in all trajectories.

In our previous studies,^30,31^ we used a constant polarizability over the nuclear space and all coherences contributed to the TRUECARS signal according to their magnitude with no further selectivity. This approximation holds when only two electronic states are involved, but as soon as more than one electronic transition is involved, the relative transition polarizability strengths determine the magnitude of the individual contributions to the total signal. This means that while the coherences themselves could be equally strong, the coherence associated with the higher transition polarizability will dominate the TRUECARS signal. We display the TRUECARS signal calculated with geometry-independent polarizability over the nuclear space in Fig. S2.† We find that the signal is particularly strong at 0 to 25 fs for S_3_/S_2_ and 90 to 100 fs for S_2_/S_1_. This signal looks more sensitive to the timing of the CIs, but the relative transition polarizability strengths determine the observed signal shown in Fig. 2a. This is more evident in trajectory 3 (Fig. S3†). The total coherence magnitude is maintained between 0 and 60 fs (Fig. S3d†), but the TRUECARS signal is strongest between 40 fs and 60 fs (Fig. S3a†) due to the large transition polarizability (Fig. S3c†). If geometry independent polarizabilities were used, the TRUECARS signal would be equally strong between 0 fs and 60 fs as shown in Fig. S3b.† This implies that, for systems undergoing multiple CIs, both the relative strength of the polarizabilities and the topologies of potential energy surfaces influence the signal and need to be properly accounted for in the simulations.

The integrated TRUECARS spectrogram reveals the energy splitting distribution between electronic states involved in the coherence.^28^ In turn, a transient energy splitting is encoded in the temporal gain/loss oscillations in the TRUECARS signal at a given Raman shift (*ω*_r_). We display the integrated frequency resolved optical-gating (FROG)^58^ spectrogram, given by eqn (7), in Fig. 2i. The spectrogram between 0 and 25 fs is distributed between 0.3 and 0.7 eV, representing the energy splitting between S_2_ and S_3/4_ during the first CI passage. The S_2_/S_1_ CI passage is captured from 30 to 50 fs. It is more evident in Fig. S4a,† where the FROG spectrogram reveals the energy splitting of CI at Raman shift *ω*_r_ = 0.02 eV. An increasing energy splitting between S_1_ and a higher excited state can be observed where the spectrogram evolves from 1 to 2 eV after 50 fs (Fig. S4b and c†). The frequency profile of the spectrogram maps the energy gap between the relevant states in Fig. 2d.

The time-evolving geometric features are displayed in Fig. 2j. These include the bond length (C_1_–C_3_, top panel), dihedral angle (C_1_ to C_4_, middle panel), and bond length alternation (0.5(b_15_ + b_48_) − b_18_, bottom panel) over time (the atomic labels are given in Fig. S6†). Earlier, we have used the same parameters to monitor the non-adiabatic passage of COT using semiempirical trajectory surface hopping dynamics.^56^ Based on the Franck-Condon approximation, the molecular geometry starts from the non-planar S_0_ minimum conformation with different C_1_–C_5_*versus* C_4_–C_8_ bond lengths, and then approaches those at planar S_1_ minimum geometry with equalized bond lengths. Indeed, the dihedral angle approaches 180°, and atomic distance increases to 3.6 Å as the population is transferred to the S_1_ state.

Previously, we had demonstrated that the coherence contribution to the TRXD signal can image the evolving electron densities during the CI passage, which is characterized by its phase oscillation between gain and loss along the temporal axis.^29,49^ The temporal oscillation showed the strongest intensities during the CI passage and the observed phase change corresponds to real-space phase changes of electron density as the molecule crosses the CI. The coherence contribution involves mixed elastic and inelastic scattering events, where the latter involves only a single active electron transition. Hence it is weak and buried under the stronger state densities, where all electrons contribute to the signal. We had suggested extraction of the information by observation at higher momentum transfer;^49^ however, it only works for systems where the transition density is more localized in real space (high ***q***) compared to the delocalized state densities (low ***q***). The valence excited states in COT, where an electron in an occupied π orbital is promoted to an unoccupied π* orbital, exhibit delocalized electron densities (Fig. S5†), and thus, the hard X-rays are not beneficial in COT. A frequency-resolved diffraction set-up,^59^ can be alternatively used since the coherences oscillate faster than the populations. In those studies, we had assumed very short waves (more than 20 keV), which are currently unavailable, and convolute the temporal resolution, rendering the experiment difficult. Currently, a new superconducting accelerator at the European X-ray Free Electron Laser in Hamburg and the Stanford Linear Coherent Light Source is being developed to provide up to 25 keV of photon energy, which may enable such frequency-resolved diffraction measurement. A development of large free electron laser facilities will enable direct observation of the evolving coherence electron densities in the future.

**Fig. 3:**
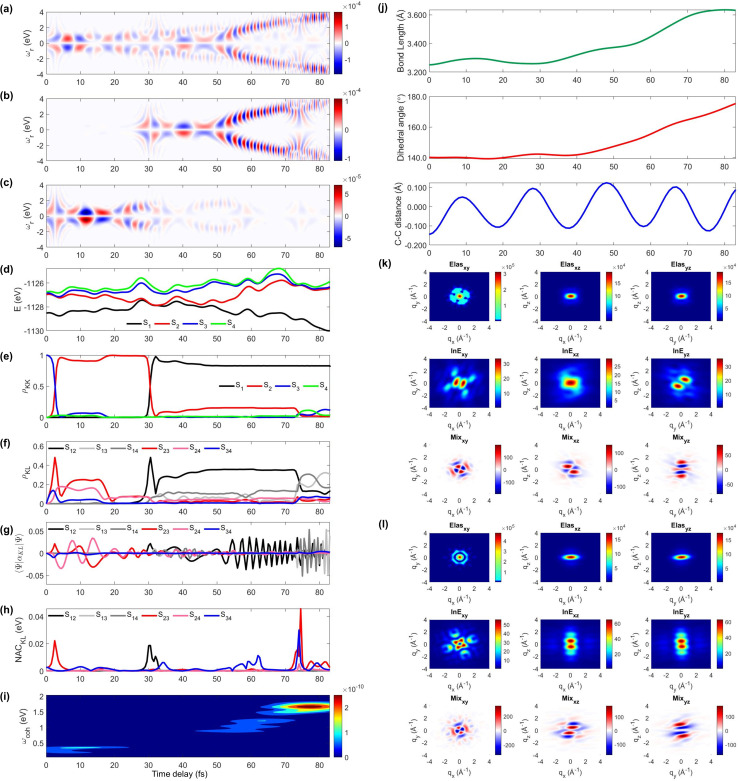
The TRUECARS and TRXD signals and relevant molecular properties in trajectory 1. The frequency-dispersed TRUECARS signal *S*(*ω*_r_, *T*), for (a) total, (b) S_2_/S_1_ coherence, and (c) S_3_/S_2_ coherence. (d) Potential energy surfaces of the excited states. (e) Population in the electronic states. (f) Coherence magnitude *ρ*_*KL*_. (g) The expectation value of the polarizability operator. (h) Nonadiabatic coupling magnitude for each coherence *ρ*_*KL*_. (i) Integrated FROG spectrogram. (j) Time evolution of the molecular geometry, bond length (top), dihedral angle (middle), and bond alternation (bottom). (k) Two-dimensional TRXD scattering pattern at *T* = 1 fs. (l) Same as (k) but at *T* = 83 fs.

**Fig. 4:**
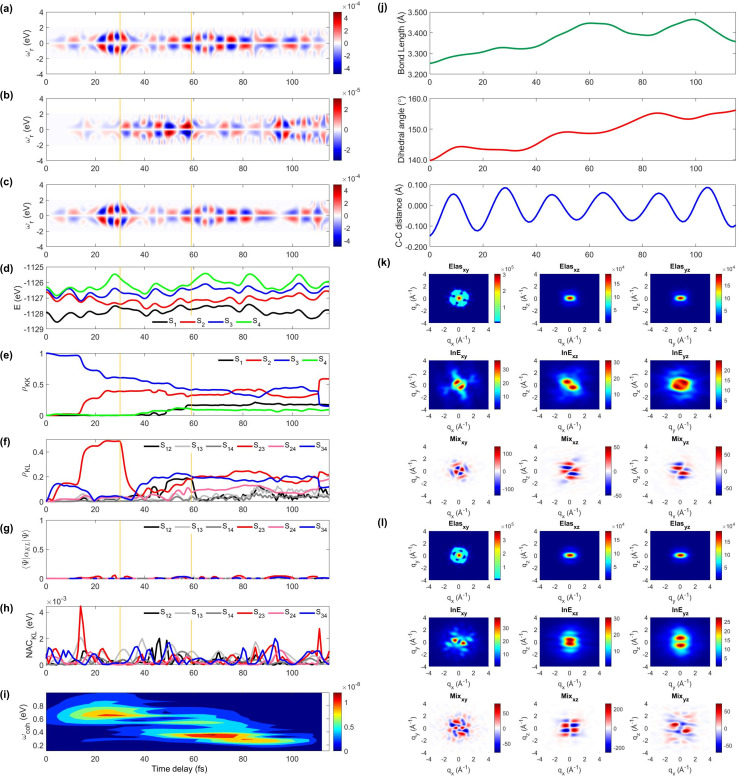
Same as Fig. 3 but for trajectory 2. The yellow vertical lines mark the cloning events. The 2D TRXD pattern shown in (l) is plotted at *T* = 115 fs.

The state densities are virtually identical for the ground state and excited state, and sensitive to the molecular conformation and electron densities. Hence, we expect that TRXD can be used to monitor the change in the molecular conformation (non-planar to planar) and aromaticity (localized electron densities at the double bond to delocalized densities). We display a two-dimensional (2D) TRXD signal in Fig. 2k and l. Note that the 2D patterns shown in Fig. 2–4 are imaged only with the valence electron densities since the semiempirical AIMC-NEXMD calculations use basis functions composed of only valence electrons. For comparison, we display the 2D pattern of the TRXD signal for S_0_ and S_1_ optimal geometries in Fig. S7,† calculated with CASSCF(8e/8o), involving all π and π* orbitals in the 6-31G* basis set. The elastic scattering pattern projected on the *xy* plane shows the localized double bond features in the S_0_ minimum. In contrast, the pattern exhibits well delocalized electron density over the entire ring in the S_1_ minimum conformation, as the molecular geometry becomes planar and all valence bonds are equalized. On comparison, the 2D pattern projected on the *xz* (Elas_xz, Fig. S7a/b† top middle panel) and *yz* pattern (Elas_yz, Fig. S7a/b† top right panel) is less sensitive but we observe an elongated pattern in the S_1_ minimum conformation compared to the non-planar conformation. We observe that the signal shows similarity with that of the S_0_ minimum in the beginning (at 1 fs, Fig. 2k) but ends with a pattern (at 250 fs, Fig. 2l) similar to that of the S_1_ minimum.

The inelastic scattering contribution from electronic populations (middle panels) or mixed elastic/inelastic scattering contribution from electronic coherence (bottom panels) does not exactly match with those in Fig. S7,† because they are calculated without considering the population (wave function coefficients) of the adiabatic states. It is not straightforward to directly compare them. Nevertheless, we note that the coherence contribution exists from 1 fs as the molecule enters the S_3_/S_2_ CI immediately. Their phase oscillations along the temporal axis could also be used to directly monitor the CI passage.

On a side note, we show that the TRXD signal can also be used to track the following S_1_ → S_0_ dynamics, owing to the different conformations of two CIs and photoproducts. We adopted their optimized geometry from the previous study^8^ and display their static 2D XRD pattern, projected on the *xy* plane in Fig. S6.† We find that the CI_b_ (Fig. S6c†) and its main product SBV (Fig. S6e†) exhibit sufficiently different XRD patterns compared to the CI_st_ (Fig. S6d†) and original COT (Fig. S6a†). The presented and discussed 2D TRXD signal may only be accessible by simulation, if the alignment of COT is not achievable.

Next, we explore two individual trajectories illustrating very different molecular dynamics scenarios. This is only possible in simulations, since only the ensemble averaged signal is observed in the experiments. However, exploring individual trajectories do help in understanding the entire molecular physics. We first describe trajectory 1, a representative scenario, where most (83%) of the populations ends with S_1_ within 100 fs without cloning events. A molecule enters the S_3_/S_2_ CI region with an immediate population transfer from S_3_ to S_2_ (Fig. 3e), and thus the TRUECARS signal shows up from the beginning (Fig. 3a and c). S_3_ and S_2_ then evolve differently (S_2_ approaches the second CI), their energy splitting increases, and the TRUECARS signal (Fig. 3c) shows faster oscillation at 20 to 30 fs than 10 to 20 fs. The second CI is reached (by nuclear wavepacket) at 30 fs, when the major population transfer between S_2_ and S_1_ is facilitated (Fig. 3e) by their large NAC (Fig. 3h). Then, the TRUECARS signal frequencies are shifted to larger Raman shifts (Fig. 3b) as the electronic energy gap between the involved states, S_1_*vs.* S_2/3/4_, increases, while S_1_ is stabilized. Note that there is no finite population transfer between S_2_ and S_1_ (after CI), but their coherence (Fig. 3f) is maintained. The signal gets even stronger as the expectation value of the transition polarizability (Fig. 2f) remains strong in this region.

The time-evolving geometric features and the 2D TRXD signal displayed in Fig. 2j–l, show that the molecule ends with planar and aromatic geometry at 83 fs, as most of the population ends with S_1_. Note that the simulation is terminated at 83 fs due to the fact that the trajectory has reached the region where the energy gap between S_1_ and S_0_ is smaller than 0.1 eV (assumed to be S_0_/S_1_ CI, see the Method section below).

Next, we illustrate an extreme opposite scenario, where several cloning events happen and the non-adiabatic simulation is terminated before reaching the S_1_ minimum (Fig. 4). In this scenario, the S_3_ state, which decays immediately in trajectory 1, survives until 110 fs (Fig. 4e). The major population transfer between S_2_ and S_3_ states occurs at 10 fs, slower than the immediate occurrence in trajectory 1. The corresponding TRUECARS signal becomes strongest between 20 and 40 fs when the S_2_ state is significantly populated and the coherence *ρ*_23_ (and thereby the expectation value of transition polarizability) is large (Fig. 4f). After 20 fs, the S_3_ and S_2_ states evolve differently, *ρ*_23_ decreases, and their energy gap increases. Eventually, at 30 fs, mean-field theory breaks down and a cloning event occurs to describe the different evolutions of the S_2_ and S_3_ states separately.

Another cloning event happens at 60 fs, when the major population transfer between the S_2_ and S_1_ ends (Fig. 4e). Due to the finite energy splitting between them (Fig. 4d) and small NAC (Fig. 4h), the S_1_ state is not significantly populated. Hence, the TRUECARS signal augmented by the S_2_/S_1_ coherence (Fig. 4b) is an order of magnitude weaker than that from the S_3_/S_2_ coherence (Fig. 4c). We find the latter dominates the total signal as the 〈*Ψ*_2_(*t*)|***α***_S_2_S_3__|*Ψ*_3_(*t*)〉 transition polarizability is strongest (Fig. 4g). The integrated FROG spectrogram shown in Fig. 4i well describes the increasing energy splitting between S_2_ and S_3_ between 20 and 40 fs, and the decreasing pattern thereafter.

We find that the relevant geometric features do not converge to those of the S_1_ minimum conformation at the end of the dynamics simulation (115 fs) because the majority of the population stays in the S_2_ state, which has a boat-like non-planar conformation, similar to the S_0_ minimum. The dihedral angle is larger than 20° and the bond length is maintained shorter than that of the delocalized ring. Thus, the 2D elastic scattering pattern projected on the *xy* plane at 115 fs shown in Fig. 4l, maintains the localized double bond feature, when compared to Fig. 4k at 1 fs.

Examination of all 98 (for TRUECARS) or 57 (for TRXD) trajectories shows highly diverse scenarios. The two trajectories discussed above are exemplary cases that contribute to the total ensemble. The consequences of the other non-adiabatic dynamics trajectories are placed in between those of the above-mentioned typical cases. Overall, we demonstrated that AIMC dynamics successfully describes the ultrafast S_3_ to S_1_ relaxation of excited states, despite the diverse evolution of individual trajectories. Note again, that only an ensemble averaged signal can be observed in the experiments.

The implementation of the proposed TRUECARS and TRXD experiments requires precise phase control between narrow and broad pulses, and the alignment of COT molecules perpendicular to the propagation of the X-ray probe pulse, respectively. Extracting coherence information in the TRXD signal requires a very hard X-ray beam, which is under development. Once achieved, the timing of the CI passages and the energetic nature of vibronic coherences can be captured by using the TRUECARS spectrogram at different Raman shifts. The evolving electron densities and molecular conformation could be tracked by using TRXD signals. The signal is off-resonant with any molecular transition; it does not require any specific core property, and directly reveals the coherences between valence electronic states.

## 3 Conclusions

We have carried out an AIMC molecular dynamics simulation to study non-adiabatic dynamics of photoexcited cyclooctatetraene, tracing its internal conversion from the bright S_3_ to the dark S_1_ state. The resulting excited state lifetime shows an excellent agreement with experiment, demonstrating an adequate characterization of the molecular photophysics by the semi-empirical multi-configurational Ehrenfest approach. The vibronic coherences created at the two major CI passages persist across the ensemble averaging over 98 trajectories and are well captured by the TRUECARS signal at different Raman shifts. We demonstrated that TRUECARS and TRXD in combination can distinguish between different CIs explored during the photoinduced dynamics. Changes in the molecular aromaticity, as well as non-planar to planar geometrical dynamics, are directly resolved in the signals. The signals combined with a semiempirical nonadiabatic molecular dynamics protocol thereby provide accurate temporal, structural and energetic profiles of the CI pathway that could reveal novel chemical design opportunities and control knobs for photochemical reactions.

## 4 Methods

The excited state non-adiabatic dynamics of COT is calculated using the *ab initio* multiple cloning (AIMC)^60,61^ approach implemented in the non-adiabatic excited state molecular dynamics (NEXMD) package.^62^ This is an extension of the Multiconfigurational Ehrenfest (MCE)^63^ method, which follows the spirit of the a*b initio* multiple spawning (AIMS) approach,^64^ allowing bifurcations of the molecular wave function in the nuclear configuration space thus naturally accounting for decoherences. Details about the connection between these two similar approaches can be found elsewhere.^65^ For AIMC, ensembles of individual Ehrenfest trajectories (clones) are used as basis functions to represent the quantum wave function of electrons and nuclei |*Ψ*(*t*)〉:^66^1
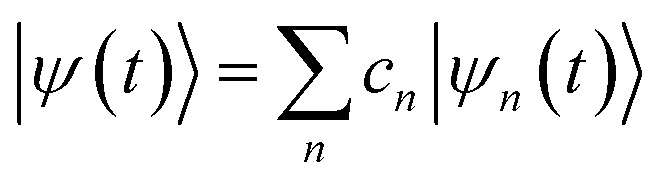


Each configuration *ψ*_*n*_(*t*) is factorized into a nuclear part *χ*_*n*_(*t*) and orthonormalized adiabatic multi-configuration electronic eigenfunctions *ϕ*^(*n*)^_*I*_:2
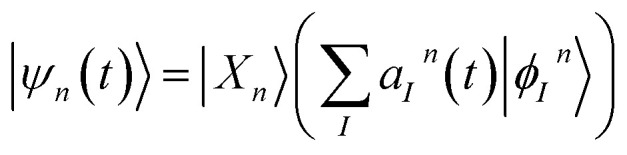


The nuclear wavepacket *χ*_*n*_(*t*) in each configuration is given by a Gaussian function centered at the Ehrenfest trajectory.

Population transfer between electronic states can occur during CI passages, where the Born Oppenheimer approximation breaks down and the motions of electrons and nuclei are strongly coupled. The original nuclear wave packet branches into multiple parts, where the excess energy follows different relaxation pathways, each dominated by a single adiabatic state. In such cases, Ehrenfest trajectories evolving on an average potential energy surface can lead to unphysical nuclear motions. AIMC recognizes these cases and replaces the original Ehrenfest trajectory configuration with two new configurations and coefficients, each evolving along its own distinct mean-field. This splitting is denoted as a cloning event, which allows to naturally account for decoherence of vibronic wavepackets evolving on the sufficiently different potential energy surfaces. More details of the AIMC method and its implementation can be found in ref. ^67^ and ^68^.

The coherences between electronic states are given by:3
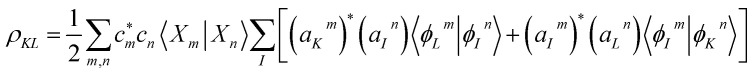


The phases of both the electronic and the nuclear parts of the molecular wave function are accounted for when calculating the vibronic coherence magnitude *ρ*_*KL*_.

The TRUECARS signal is finally given by:^26^4
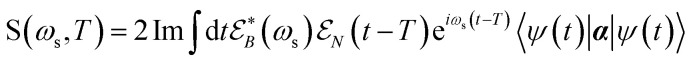
where “Im” denotes the imaginary part, 
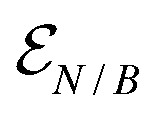
 is a hybrid narrow (2 femtosecond)/broadband (500 attosecond) Gaussian pulse envelope (Fig. 1a), *ω*_s_ is the central probe frequency, and *T* is the time delay between the pump and the probe. The expectation value of the transition polarizability 〈*Ψ*(*t*)|***α***_*KL*_|*Ψ*(*t*)〉 is given by5
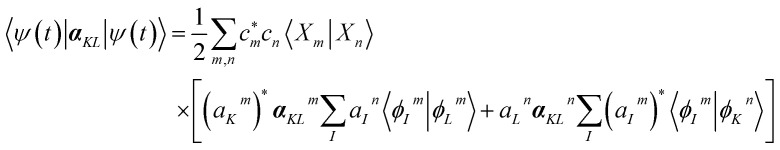


The transition polarizability ***α***_*KL*_ is calculated from the transition charge density, ***σ***_*KL*_, where6
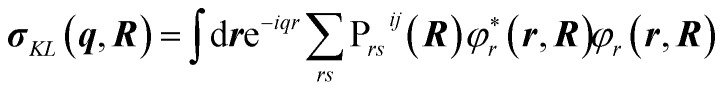
using the state charge density matrices P_*rs*_^*ij*^, and the basis set of atomic orbitals *φ*_r_(***r***). Populations do not contribute to the signal, since ***α***_*KK*_ is zero along the diagonal, and only the transition polarizabilities (off-diagonal elements) between electronic states are finite. The TRUECARS signal is calculated for a randomly oriented ensemble by averaging over the *x*, *y*, and *z* axes. We shall display the frequency resolved optical-gating (FROG) spectrogram of the TRUECARS signal given by ref. ^58^, by convolving a temporal trace *S*(*t*) at a constant *ω*_r_, with a Gaussian gating function *E*_gate_(*t*) with a full width at half-maximum (fwhm) of 0.484 fs,7
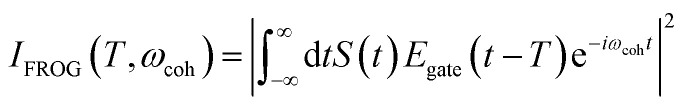
*S*(*T*) oscillates with frequencies that correspond to the energy splitting between the relevant vibronic coherences, and the FROG spectrogram reveals the transient energy splitting along the trajectory. The FROG spectrograms are scanned and integrated over a negative Raman shift (*ω*_r_ < 0) window to capture the evolution of the signal away from *ω*_r_ = 0.

The gas phase (single-molecule) TRXD signal of a sample with *N* non-interacting molecules reads^33,69^8
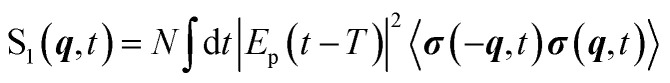
where9
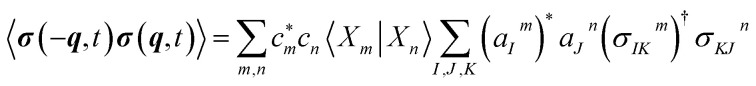
where 〈*ϕ*_*I*_^*m*^|***σ***(−***q***,*t*)|*ϕ*_*K*_^*m*^〉 = (*σ*_*IK*_^*m*^)^†^ and 〈*ϕ*_*K*_^*n*^|***σ***(***q***,*t*)|*ϕ*_J_^*n*^〉 = *σ*_*KJ*_^*n*^. We refer the reader to the ESI† for the derivation more detail.

AIMC simulations of COT have been performed at constant energy using a 0.05 fs time step. The initial conformational structures were sampled from a 520 ps ground-state adiabatic molecular dynamics trajectory in a vacuum using a Langevin thermostat at 300 K with a 0.1 fs time step.^56^ Following a 20 ps equilibration period, 100 snapshots of geometries and velocities were harvested every 10 ps and used as the initial conditions for the AIMC non-adiabatic molecular dynamics simulation. Nuclear dynamics were simulated for all 42 nuclear degrees of freedom, from the initial excited S_3_ state populated by an impulsive excitation. Note that S_1_ is a dark state and S_2_ and S_3_ are both bright degenerate states, but the latter has 6 times larger oscillater strength than S_2_ during the ground state dynamics (see Fig. 1e in ref. ^56^). The S_4_ state is located 0.47 eV higher than the S_2/3_ state, and thus, one can safely exclude the excitation to the S_4_ state by using a spectrally narrow enough optical pump pulse. If we follow what Levine *et al.* had done in their butadiene work,^70^ our initial condition should shift 14.3% of the population to S_2_ and the remaining major population to S_3_. We do not expect substantial differences in TRUECARS and TRXD signals, but the relaxation timescale of COT could be a bit faster.

Excited state properties (*e.g.*, energies, gradients, and non-adiabatic couplings) are calculated on-the-fly at the configuration interaction single (CIS) level of theory using the semiempirical Austin model 1 Hamiltonian.^71^ The non-adiabatic transitions to the ground state near S_0_/S_1_ CI have an inherent superposition or multireference character which cannot be properly described with single-reference CIS, time-dependent Hartree Fock or time-dependent density functional theory due to an incorrect description of the topology of the CI near the crossing (phase factors, *etc*).^72–74^ An alternative approach is the “Open-GS” method^75,76^ that enforces such a transition to the ground state when the energy gap between the ground and excited states is smaller than a certain threshold. Hence, we set up a 0.1 eV threshold value for S_1_/S_0_ CI description so that the AIMC simulation is terminated, once one of the clones reaches this point. Finally, 98 independent trajectories were averaged for further TRUECARS signal analysis, whereas 57 trajectories were used for the TRXD signal. We found that TRXD computations require extremely large data storage (*e.g.*, each trajectory occupies around 66 GB, and the largest one takes up 200 GB) but averaging over 57 trajectories is enough to get converged results for TRXD signals. The computations of TRUECARS signals are not subjected to such computational cost, and we averaged over all 98 trajectories.

The use of transition polarizabilities, though computationally demanding, gives more accurate results compared with our previous work, where we set ***α***_*KL*_ to be constant (geometry-independent) over the nuclear space, thereby reducing 〈*Ψ*(*t*)|***α***|*Ψ*(*t*)〉 to the overlap between the involved electronic states (vibronic coherence magnitude).^30,31^

## Data availability

All study data are included in the article and/or ESI.†

## Author contributions

Y. N. calculated the X-ray signals and wrote the manuscript. H. S. simulated non-adiabatic molecular dynamics and wrote the manuscript. V. M. F. and S. F.-A. derived the TRUECARS and TRXD signals. D. K. helped in computing and analyzing the TRUECARS signal and its spectrogram. J. Y. L. and S. T. supervised the project and wrote the manuscript. M. G. suggested the cyclooctatetraene molecule and wrote part of introduction. S. M. designed and supervised the project and wrote the manuscript.

## Conflicts of interest

There are no conflicts to declare.

## Supplementary Material

SC-014-D2SC04335A-s001
