# Supplementary material for: Monitoring vibronic coherences and molecular aromaticity in photoexcited cyclooctatetraene with an X-ray probe: a simulation study
Source: Chem Sci. 2023 Feb 14;14(11):2971–82. doi: 10.1039/d2sc04335a (PMC10016608; doi:10.1039/d2sc04335a)
Supplement: SC-014-D2SC04335A-s001 [file SC-014-D2SC04335A-s001.pdf]

Electronic Supplementary Material (ESI) for Chemical Science.
This journal is © The Royal Society of Chemistry 2023

-Supporting Information-
Monitoring Vibronic Coherences and Molecular Aromaticity in Photoexcited Cyclooctatetraene with X-ray Probe: A Simulation Study

Yeonsig Nam[1,*,†], Huajing Song[2,†], Victor M. Freixas[3], Daniel Keefer[1], Sebastian Fernandez-Alberti[3], Jin Yong Lee[4*], Marco Garavelli[5], Sergei Tretiak[2], and Shaul Mukamel[1*]

[1]Department of Chemistry, University of California, Irvine, California 92697-2025, United States
[2]Physics and Chemistry of Materials, Theoretical Division, Los Alamos National Laboratory, Los Alamos, New Mexico 87545, USA.
[3]Departamento de Ciencia y Tecnologia, Universidad Nacional de Quilmes/CONICET, B1876BXD, Bernal, Argentina
[4]Department of Chemistry, Sungkyunkwan University, Suwon 16419, Korea
[5]Dipartimento di Chimica Industriale "Toso Montanari,", Universita' degli Studi di Bologna, I-40136 Bologna, Italy
*yeonsign@uci.edu, jinylee@skku.edu, smukamel@uci.edu
†These authors contributed equally to this work

November 16, 2022

Contents

1 Signal Derivation **2**
1.1 TRUECARS signal 2
1.2 Time-resolved X-ray Diffraction 3

2 Loop Diagram Rules **4**

3 Supplementary Figures **4**

1 Signal Derivation

1.1 TRUECARS signal

The TRUECARS signal is finally given by: [1]

$$S(\omega_s, T) = 2\mathrm{Im}\int \mathrm{d}t\mathcal{E}_B^*(\omega_s)\mathcal{E}_N(t-T)e^{i\omega_s(t-T)}\langle\Psi(t)|\boldsymbol{\alpha}|\Psi(t)\rangle \tag{1}$$

where "Im" denotes the imaginary part, $\mathcal{E}_{N/B}$ is a hybrid narrow (2 femtosecond)/broadband (500 attosecond) Gaussian pulse envelope (Figure 1a), ω_s is the central probe frequency, T is the time delay between the pump and the probe. The relevant time-dependent material quantity for this signal is the expectation value of the polarizability operator $\boldsymbol{\alpha}$ that can be calculated by

$$\boldsymbol{\alpha}_{KL} = |\phi_K^R\rangle\boldsymbol{\alpha}_{KL}^R\langle\phi_L^R| \tag{2}$$

the corresponding expectation value is given by:

$$\langle\Psi(t)|\boldsymbol{\alpha}_{KL}|\Psi(t)\rangle = \sum_{n,m} c_m^* c_n\langle\chi_m|\chi_n\rangle\sum_{I,J}(a_J^m)^* a_I^n\langle\phi_J^m|\boldsymbol{\alpha}_{KL}|\phi_I^n\rangle \tag{3}$$

By plugging Eqn 2 into Eqn 3, we get:

$$\langle\Psi(t)|\boldsymbol{\alpha}_{KL}|\Psi(t)\rangle = \sum_{n,m} c_m^* c_n\langle\chi_m|\chi_n\rangle\sum_{I,J}(a_J^m)^* a_I^n\langle\phi_J^m|\phi_K^R\rangle\boldsymbol{\alpha}_{KL}^R\langle\phi_L^R|\phi_I^n\rangle \tag{4}$$

We now invoke the approximation (see Eqn 24 in Ref [2])

$$\langle\phi_J^m|\phi_K^R\rangle\boldsymbol{\alpha}_{KL}^R\langle\phi_L^R|\phi_I^n\rangle \approx \frac{1}{2}\left[\langle\phi_J^m|\phi_K^m\rangle\boldsymbol{\alpha}_{KL}^m\langle\phi_L^m|\phi_I^n\rangle + \langle\phi_J^m|\phi_K^n\rangle\boldsymbol{\alpha}_{KL}^n\langle\phi_L^n|\phi_I^n\rangle\right] \tag{5}$$

Using the orthonormality of the adiabatic basis:

$$\langle\phi_J^m|\phi_K^m\rangle = \delta_{JK}^m, \langle\phi_L^n|\phi_I^n\rangle = \delta_{LI}^n \tag{6}$$

we get:

$$\langle\phi_J^m|\phi_K^R\rangle\boldsymbol{\alpha}_{KL}^R\langle\phi_L^R|\phi_I^n\rangle \approx \frac{1}{2}\left[\langle\phi_L^m|\phi_I^n\rangle\boldsymbol{\alpha}_{KL}^m\delta_{JK}^m + \boldsymbol{\alpha}_{KL}^n\delta_{LI}^n\langle\phi_J^m|\phi_K^n\rangle\right] \tag{7}$$

Plugging Eqn 7 into Eqn 4, simplifying the Kronecker deltas, and substituting the index J into I gives:

$$\langle\Psi(t)|\boldsymbol{\alpha}_{KL}|\Psi(t)\rangle = \frac{1}{2}\sum_{m,n} c_m^* c_n\langle\chi_m|\chi_n\rangle\left[(a_K^m)^*\boldsymbol{\alpha}_{KL}^m\sum_I a_I^n\langle\phi_L^m|\phi_I^n\rangle + a_L^n\boldsymbol{\alpha}_{KL}^n\sum_I(a_I^m)^*\langle\phi_I^m|\phi_K^n\rangle\right] \tag{8}$$

The transition polarizability $\boldsymbol{\alpha}_{KL}$ is calculated from the transition charge density, σ_{KL}, where

$$\sigma_{KL}(\boldsymbol{q},\boldsymbol{R}) = \int dre^{-i\boldsymbol{qr}}\sum_{rs}P_{rs}^{ij}(\boldsymbol{R})\varphi_r^*(\boldsymbol{r},\boldsymbol{R})\varphi_r(\boldsymbol{r},\boldsymbol{R}), \tag{9}$$

using the state charge density matrices P_{rs}^{ij}, and the basis set of atomic orbitals $\varphi_r(\boldsymbol{r})$. Populations do not contribute to the signal, since $\boldsymbol{\alpha}_{KK}$ is zero along the the diagonal, and only the transition polarizabilities (off-diagonal elements) between electronic states are finite. The effective polarizability value is computed from the transition charge density at 200 eV. For example, the effective transition polarizability along z-axis is calculated, $\boldsymbol{\alpha}_{\text{eff}}(\boldsymbol{q}) = \boldsymbol{\sigma}(\boldsymbol{q})$ at $q_x = 0$, $q_y = 0$, and $q_z = |k_z| = \omega_s/c$, where ω_s is the probe carrier frequency and c is the speed of the light, 137.036 a.u.. The TRUECARS signal is calculated for a randomly oriented ensemble by averaging over the x, y, and z axes. We shall display the Frequency resolved optical-gating (FROG) spectrogram of the TRUECARS signal given by Ref[3], by convolving a temporal trace $S(t)$ at a constant ω_r, with a Gaussian gating function $E_{\text{gate}}(t)$ with a full width at half-maximum (fwhm) of 0.484 fs,

$$I_{\text{FROG}}(T, \omega_{\text{coh}}) = \left| \int_{-\infty}^{\infty} \mathrm{d}t S(t) E_{\text{gate}}(t-T) e^{-i\omega_{\text{coh}} t} \right|^2. \tag{10}$$

$S(T)$ oscillates with frequencies that correspond to the energy splitting between the relevant vibronic coherences, and the FROG spectrogram reveals the transient energy splitting along the trajectory. The FROG spectrograms are scanned and integrated over negative Raman shift ($\omega_r < 0$) window to capture the evolution of the signal away from $\omega_r = 0$.

1.2 Time-resolved X-ray Diffraction

The gas phase (single-molecule) TRXD signal of a sample with N non-interacting molecules reads[4], [5]

$$S_1(\boldsymbol{q}, t) = N \int \mathrm{d}t |E_p(t-T)|^2 \langle \boldsymbol{\sigma}(-\boldsymbol{q}, t) \boldsymbol{\sigma}(\boldsymbol{q}, t) \rangle \tag{11}$$

where,

$$\langle \boldsymbol{\sigma}(-\boldsymbol{q}, t) \boldsymbol{\sigma}(\boldsymbol{q}, t) \rangle = \langle \Psi(t) | \boldsymbol{\sigma}(-\boldsymbol{q}, t) \boldsymbol{\sigma}(\boldsymbol{q}, t) | \Psi(t) \rangle \tag{12}$$

It can be expanded into

$$\langle \boldsymbol{\sigma}(-\boldsymbol{q}, t) \boldsymbol{\sigma}(\boldsymbol{q}, t) \rangle = \sum_{m,n} c_m^* c_n \langle \chi_m | \chi_n \rangle \sum_{I,J} (a_I^m)^* a_J^n \langle \phi_I^m | \boldsymbol{\sigma}(-\boldsymbol{q}, t) \boldsymbol{\sigma}(\boldsymbol{q}, t) | \phi_J^n \rangle \tag{13}$$

If we use the identity relation (Eqn 5):

$$\langle \boldsymbol{\sigma}(-\boldsymbol{q}, t) \boldsymbol{\sigma}(\boldsymbol{q}, t) \rangle = \sum_{m,n} c_m^* c_n \langle \chi_m | \chi_n \rangle \sum_{I,J} (a_I^m)^* a_J^n \times \langle \phi_I^m | \boldsymbol{\sigma}(-\boldsymbol{q}, t) | \phi_K^R \rangle \langle \phi_K^R | \boldsymbol{\sigma}(\boldsymbol{q}, t) | \phi_J^n \rangle \tag{14}$$

A reasonable approximation would be

$$\langle \phi_I^m | \boldsymbol{\sigma}(-\boldsymbol{q}, t) | \phi_K^R \rangle \langle \phi_K^R | \boldsymbol{\sigma}(\boldsymbol{q}, t) | \phi_J^n \rangle \approx \tag{15}$$

$$\frac{1}{2} \left[\langle \phi_I^m | \boldsymbol{\sigma}(-\boldsymbol{q}, t) | \phi_K^m \rangle \langle \phi_K^m | \boldsymbol{\sigma}(\boldsymbol{q}, t) | \phi_J^n \rangle + \langle \phi_I^m | \boldsymbol{\sigma}(-\boldsymbol{q}, t) | \phi_K^n \rangle \langle \phi_K^n | \boldsymbol{\sigma}(\boldsymbol{q}, t) | \phi_J^n \rangle \right] \tag{16}$$

however, we do not have access for the moment to the mixed matrix elements, $\langle\phi_K^m|\boldsymbol{\sigma}(\boldsymbol{q},t)|\phi_J^n\rangle$ or $\langle\phi_I^m|\boldsymbol{\sigma}(-\boldsymbol{q},t)|\phi_K^n\rangle$. A cruder approximation is

$$\langle\phi_I^m|\boldsymbol{\sigma}(-\boldsymbol{q},t)|\boldsymbol{\sigma}(\boldsymbol{q},t)|\phi_J^n\rangle \approx \sum_{K,L=1}^{4}\langle\phi_I^m|\boldsymbol{\sigma}(-\boldsymbol{q},t)|\phi_L^m\rangle\langle\phi_L^m|\phi_K^n\rangle\langle\phi_K^n|\boldsymbol{\sigma}(\boldsymbol{q},t)|\phi_J^n\rangle \tag{17}$$

which gives

$$\langle\boldsymbol{\sigma}(-\boldsymbol{q},t)\boldsymbol{\sigma}(\boldsymbol{q},t)\rangle = \sum_{m,n} c_m^* c_n\langle\chi_m|\chi_n\rangle \sum_{I,J,K,L}(a_I^m)^* a_J^n(\sigma_{IK}^m)^\dagger S_{LK}^{mn}\sigma_{KJ}^n \tag{18}$$

where, $\langle\phi_I^m|\boldsymbol{\sigma}(-\boldsymbol{q},t)|\phi_K^m\rangle = (\sigma_{IK}^m)^\dagger$, $\langle\phi_K^n|\boldsymbol{\sigma}(\boldsymbol{q},t)|\phi_J^n\rangle = \sigma_{KJ}^n$, and $\langle\phi_L^m|\phi_K^n\rangle = S_{LK}^{mn}$. Neglecting electronic wavefunction overlap may be inaccurate when the nuclear configuration m and n are far from each other. However, the nuclear overlap $\langle\chi_m|\chi_n\rangle$ should go to zero too, hence, the corresponding error should vanish.

2 Loop Diagram Rules

The loop diagram of the optical pump off-resonant X-ray probe signal is shown in Fig. 1 in main text and Fig. S1. The Diagram rules are as follows:

- Time runs along the loop clockwise from bottom left to bottom right.
- Each field interaction is represented by an arrow, which either points to the right (photon annihilation and excitation of the molecule) or to the left (photon creation and de-excitation of the molecule).
- Free evolution periods on the left branch indicate forward propagation in real time, and on the right branch to backward propagation, respectively.
- The last field interaction is the detected photon mode. In addition, the grey bar represents the period of free evolution.

3 Supplementary Figures

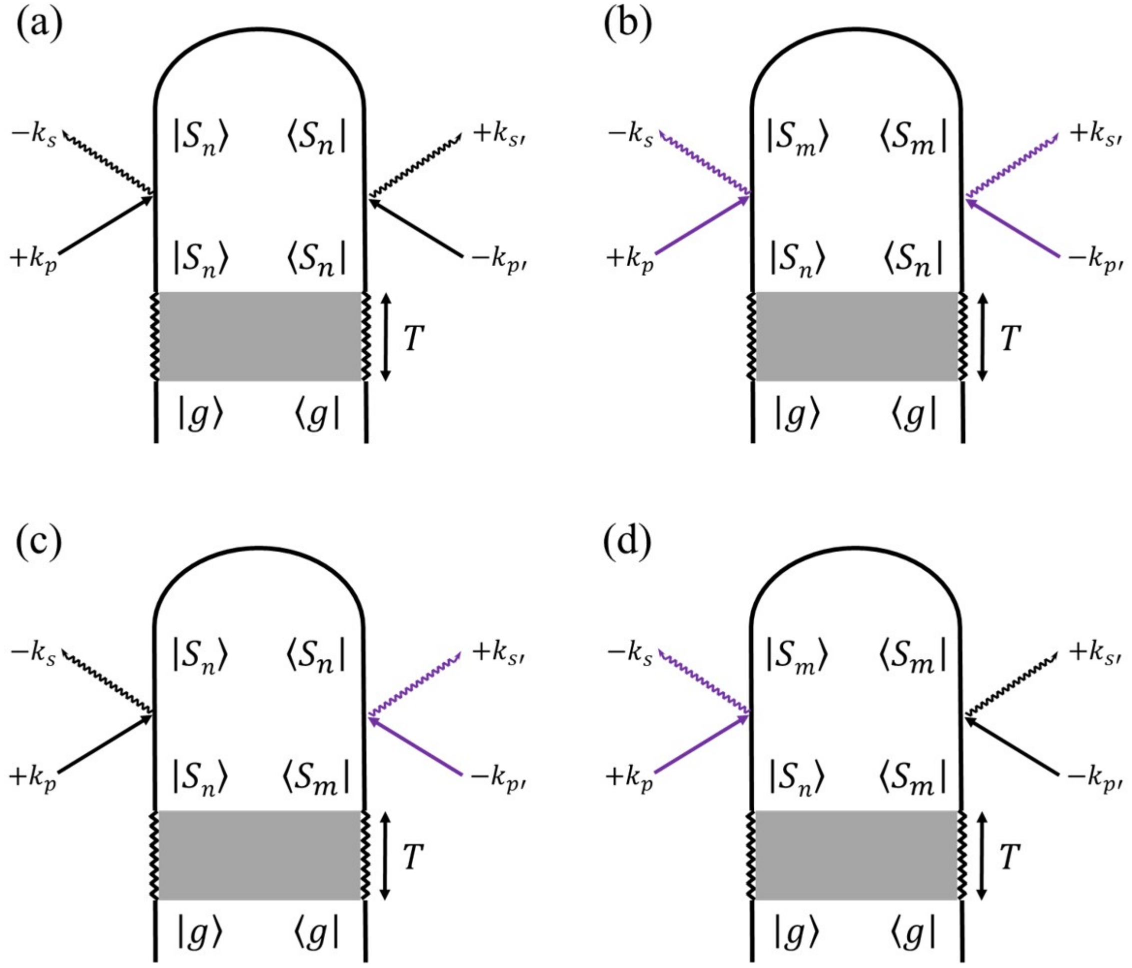

Figure S 1: Loop diagrams for single-molecule X-ray scattering process. The shaded area represents an excitation that prepares the system in S_3 state and a field-free nonadiabatic dynamics during time delay T. We denote modes of the X-ray probe pulse with p and p′, whereas s and s′ represent relevant scattering modes. Elastic scattering process are denoted by black field arrows. Inelastic processes are denoted by purple arrows. The indices n and m runs over excited states, S_1 to S_4.

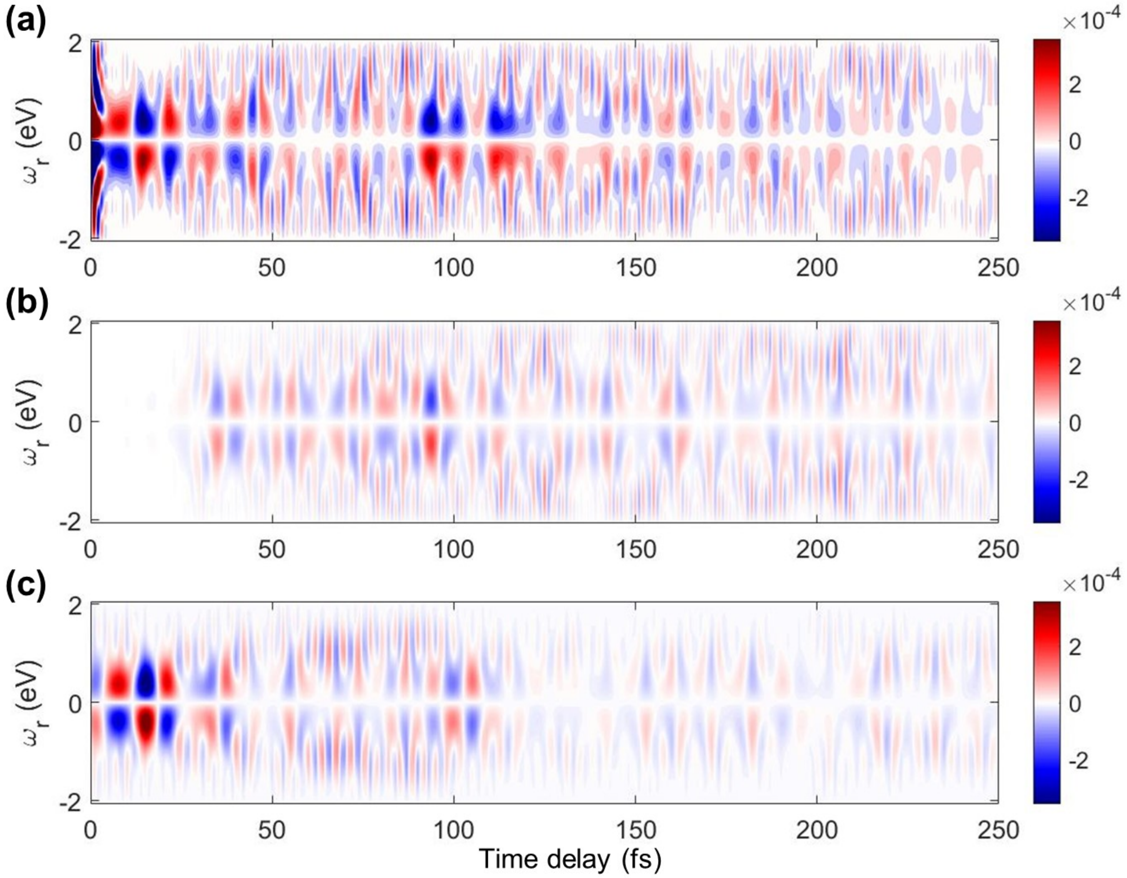

Figure S 2: The averaged (ensemble) TRUECARS signal calculated with a constant (geometry independent) transition polarizability operator, which equals to the vibronic coherence magnitude (Fig. 2f). The panel b and c separately shows TRUECARS signal for S_1/S_2 and S_2/S_3 coherence, respectively.

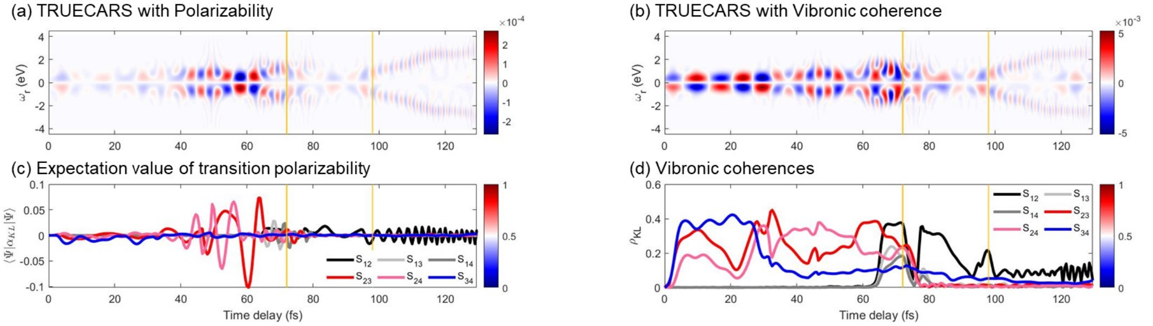

Figure S 3: The TRUECARS signal for trajectory 3. The yellow lines indicate the cloning events. The TRUECARS signal (a) calculated with the expectation value of the transition polarizability operator (c). The TRUECARS signal (b) calculated with a constant (geometry independent) transition polarizability operator, which equals to the vibronic coherence magnitude (d).

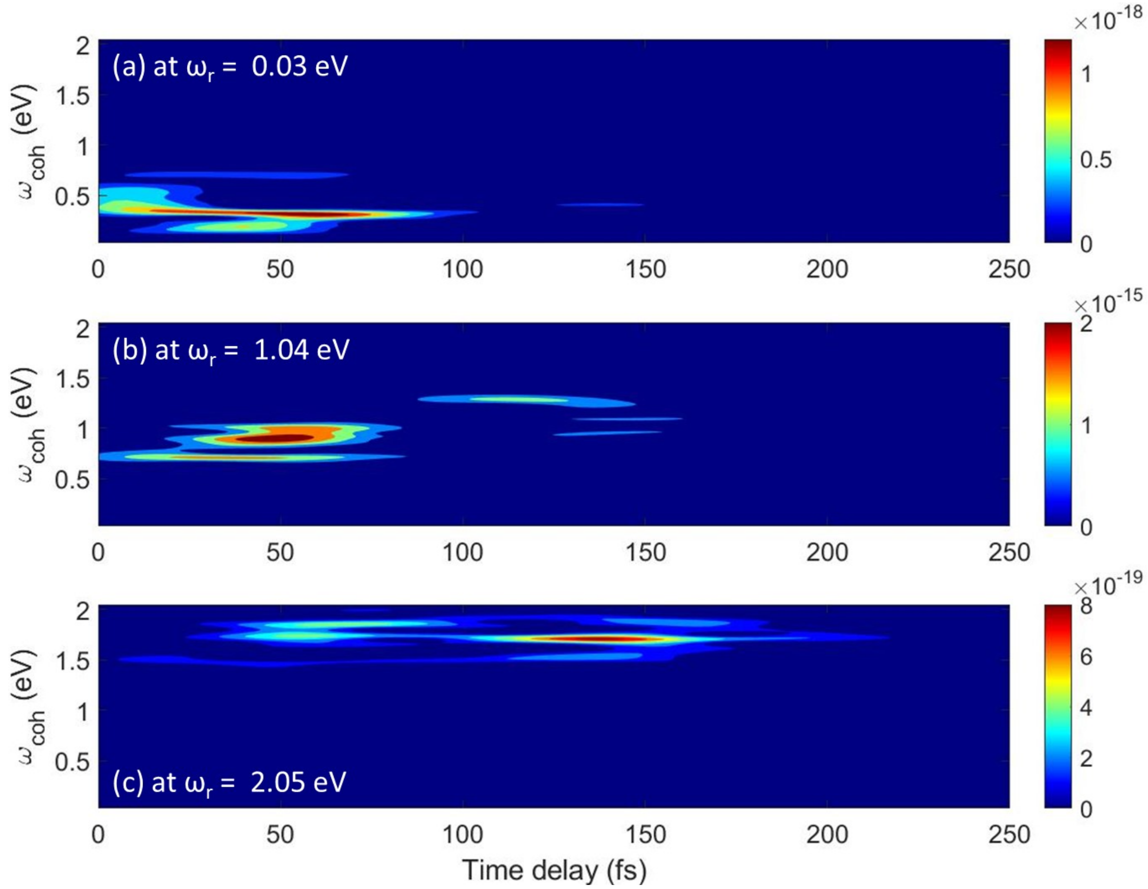

Figure S 4: Transient frequency-resolved optical-gating spectrogram (eq 10 in ESI) with signal trace taken at ω_r = (a) 0.03, (b) 1.04, and (c) 2.05 eV.

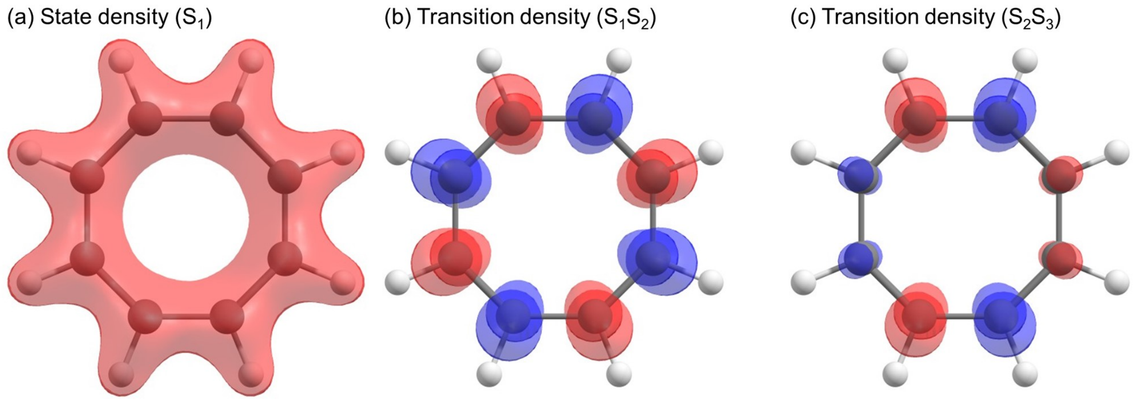

Figure S 5: Real-space charge densities of COT at S_1 minimum geometry. isovalue 0.1/0.005 for state/transition densities, respectively

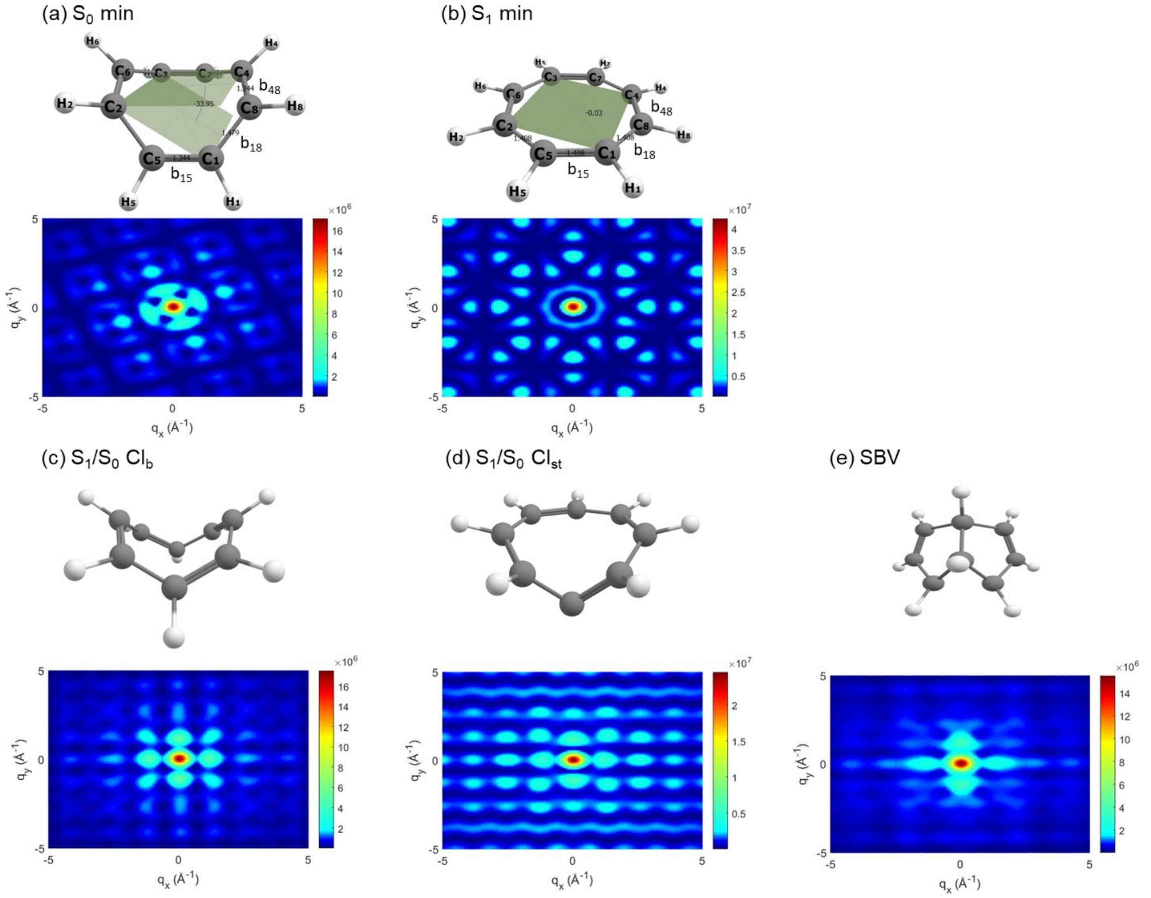

Figure S 6: The optimized geometries (from Ref [6]) of (a) S_0 minimum, (b) S_1 minimum, (c) CI_b, (d) CI_{st}, (e) SBV, and their two-dimensional TRXD elastic scattering pattern, projected on the xy plane. Calculated with CASSCF(8e/8o), involving all π and π^* orbitals at 6-31G* basis set

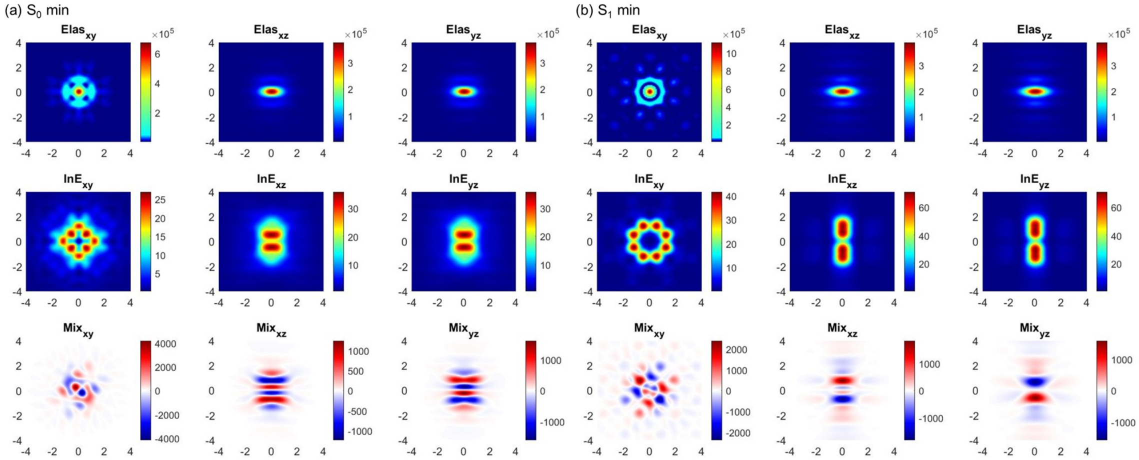

Figure S 7: The two-dimensional XRD elastic scattering pattern, projected on the xy (left), xz (middle), and yz (right) plane for elastic (top), inelastic (middle), and mixed elastic/inelastic (coherence, bottom) scattering at (a) S_0 minimum and (b) S_1 minimum geometry. Calculated with CASSCF(8e/8o), involving all π and π^* orbitals at 6-31G* basis set

References

[1] M. Kowalewski, K. Bennett, K. E. Dorfman, and S. Mukamel, "Catching conical intersections in the act: Monitoring transient electronic coherences by attosecond stimulated x-ray raman signals," *Phys. Rev. Lett.*, vol. 115, p. 193 003, 19 Nov. 2015. DOI: 10.1103/PhysRevLett.115.193003. [Online]. Available: https://link.aps.org/doi/10.1103/PhysRevLett.115.193003.

[2] S. Fernandez-Alberti, D. V. Makhov, S. Tretiak, and D. V. Shalashilin, "Non-adiabatic excited state molecular dynamics of phenylene ethynylene dendrimer using a multiconfigurational ehrenfest approach," *Phys. Chem. Chem. Phys.*, vol. 18, pp. 10 028–40, 2016.

[3] R. Trebino, K. W. DeLong, D. N. Fittinghoff, *et al.*, "Measuring ultrashort laser pulses in the time-frequency domain using frequency-resolved optical gating," *Review of Scientific Instruments*, vol. 68, no. 9, pp. 3277–3295, 1997. DOI: 10.1063/1.1148286. eprint: https://doi.org/10.1063/1.1148286. [Online]. Available: https://doi.org/10.1063/1.1148286.

[4] K. Bennett, J. D. Biggs, Y. Zhang, K. E. Dorfman, and S. Mukamel, "Time-, frequency-, and wavevector-resolved x-ray diffraction from single molecules," *The Journal of Chemical Physics*, vol. 140, no. 20, p. 204 311, 2014. DOI: 10.1063/1.4878377. eprint: https://doi.org/10.1063/1.4878377. [Online]. Available: https://doi.org/10.1063/1.4878377.

[5] K. Bennett, M. Kowalewski, J. R. Rouxel, and S. Mukamel, "Monitoring molecular nonadiabatic dynamics with femtosecond x-ray diffraction," *Proceedings of the National Academy of Sciences*, vol. 115, no. 26, pp. 6538–6547, 2018. DOI: 10.1073/pnas.1805335115. eprint: https://www.pnas.org/doi/pdf/10.1073/pnas.1805335115. [Online]. Available: https://www.pnas.org/doi/abs/10.1073/pnas.1805335115.

[6] M. Garavelli, F. Bernardi, A. Cembran, *et al.*, "Cyclooctatetraene computational photo- and thermal chemistry: A reactivity model for conjugated hydrocarbons," *Journal of the American Chemical Society*, vol. 124, no. 46, pp. 13 770–13 789, 2002, PMID: 12431107. DOI: 10.1021/ja020741v. eprint: https://doi.org/10.1021/ja020741v. [Online]. Available: https://doi.org/10.1021/ja020741v.
